# Stress indicators in dairy cows adapting to virtual fencing

**DOI:** 10.1093/jas/skae024

**Published:** 2024-01-25

**Authors:** Patricia Fuchs, Joanna Stachowicz, Manuel K Schneider, Massimiliano Probo, Rupert M Bruckmaier, Christina Umstätter

**Affiliations:** Graduate School for Cellular and Biomedical Sciences, University of Bern, 3012 Bern, Switzerland; Agroscope, Research Division Animal Production Systems and Animal Health, Grazing Systems, 1725 Posieux, Switzerland; Johann Heinrich von Thünen-Institute, Institute of Agricultural Technology, 38116 Braunschweig, Germany; Agroscope, Research Division Animal Production Systems and Animal Health, Forage Production and Grassland Systems, 8046 Zurich, Switzerland; Agroscope, Research Division Animal Production Systems and Animal Health, Grazing Systems, 1725 Posieux, Switzerland; Veterinary Physiology, Department of Clinical Research and Public Health, Vetsuisse Faculty, University of Bern, 3001 Bern, Switzerland; Johann Heinrich von Thünen-Institute, Institute of Agricultural Technology, 38116 Braunschweig, Germany

**Keywords:** animal welfare, dairy cow, electric pulse, learning behavior, pasture management, virtual fencing

## Abstract

Virtual fencing (**VF**) enables livestock grazing without physical fences by conditioning animals to a virtual boundary delimited with an audio tone (**AT**) and an electric pulse (**EP**). The present study followed the adaptation process of lactating dairy cows to a VF system with changing virtual boundaries and investigated its impact on animal welfare. Twenty cows were divided into stratified groups (2× VF; 2× electric fencing, **EF**) of five individuals. Each group grazed half-days in a separate EF paddock of comparable size during 3 d of acclimation (**P0**), followed by 21, 14, 14, and 7 d of experimental treatment (**P1 to P4**). At the start of the trial, all cows were equipped with an IceQube pedometer (Peacock Technology Ltd, Stirling, UK) and a VF collar (Nofence AS, Batnfjordsøra, Norway). During P0, cows were accustomed to their first paddock with a deactivated virtual boundary and wearing the sensors. In P1 to P4, an active virtual boundary for the VF groups, and a second EF for the EF groups was set up parallel to an outer EF within their paddock. Throughout the trial, the sensors continuously tracked cow positions and activity behavior at 15-min intervals. From P1 onwards, the VF collars additionally recorded each AT and EP per cow with a georeferenced time stamp. During P0 to P4, daily feed intake, body weight, and milk yield were recorded in the barn. A total of 26 milk samples were collected per cow to determine milk cortisol levels. Behavioral observations were conducted for 2 h on day 23 to record agonistic behaviors, vocalizations, and excretions. The total number of stimuli per cow ranged from 37 to 225 ATs (mean ± SD: 1.9 ± 3.3 per day) and 3 to 11 EPs (mean ± SD: 0.1 ± 0.7 per day) throughout the trial. The maximum number of EPs per day was 8 for an individual cow and occurred once on D1. Mean EP/AT decreased by 55% during the first three half-days of grazing and with each paddock change from 0.2 EP/AT in week 1 to 0.03, 0.02, and 0 EP/AT in weeks 4, 6, and 8, respectively. Linear and generalized mixed effects models revealed that milk yield and cortisol, feed intake, body weight, and activity and lying behavior did not significantly differ between VF and EF groups. A higher number of agonistic behaviors were observed in the VF groups when the VF system was activated. However, due to the short observation periods only few contacts were observed in total. Overall, all cows adapted to the VF system without evidence of lasting adverse effects on animal welfare.

## Teaser

We investigated the learning process of dairy cows with changing virtual fence lines, while examining stress indicators in the animals to either support or refute animal welfare concerns associated with virtual fencing (**VF**). Our study shows that dairy cows were introduced to a VF system without lasting negative effects on animal welfare, which may help to ease legal restrictions on the use of VF on farms.

## Introduction

The use of fences is an essential part of modern grazing management in Europe. Technological steps led to the first commercial virtual fencing (**VF**) system in 1973 based on an induction wire for controlling domestic cats and dogs (Umstatter, 2011). Recent innovations target livestock grazing based on Global Navigation Satellite Systems (**GNSS**), generating a digital boundary that can take any geometric shape (Anderson, 2007). A VF system consists of a collar with an integrated tracking system and a tool to administer a paired sequence of an audio tone (**AT**) and an electric pulse (**EP**) when the animal crosses the virtual boundary. The technology opens up the possibility of replacing physical fences with virtual ones. It also allows pasture boundaries to be flexibly adapted to the needs of animals and natural conditions. This can reduce manual labor, which is valuable in intensive grazing, but also in marginal areas where geography, terrain, and associated costs make conventional fencing impractical (Umstatter, 2011).

However, the use of a VF system raises concerns from an animal welfare perspective, in particular, the ability of animals to learn the concept of VF and the associated welfare implications of using EPs (Stampa et al., 2020). Firstly, unlike physical barriers, a virtual fence is not obvious to livestock. Cows rely more on visual than auditory cues, although they have a wide range of hearing that allows them to perceive high-frequency sounds in the ultrasonic range (Heffner and Heffner, 2008). McSweeney et al. (2020) showed that the number of interactions with the virtual fence increased in dairy cows in the absence of visual cues, but no further interactions occurred when visual cues were reintroduced. Second, the EP-emitting device is directly attached to the animal. The animal cannot isolate itself from aversive stimuli and may receive uncontrolled shocks in the event of technical malfunctions. Third, the presence of herd mates during learning positively influences the ability or time required for individual cattle (Colusso et al., 2020; Keshavarzi et al., 2020; McSweeney et al., 2020) and sheep (Marini et al., 2020) to associate the paired stimuli. However, associative learning is facilitated by individual exposure to VF, which in turn is difficult to implement and maintain in herded animals (Colusso et al., 2020). Fourth, not all animals in the herd may simply have the ability to learn the association of AT and EP and thus cope with the VF system. Marini et al. (2020) showed that sheep with a high proportion of EPs relative to their fence contacts avoided approaching the virtual boundary altogether, thus failing to learn the system.

In contrast, several studies have found that sheep (Marini et al., 2018a, 2018b), beef (Verdon et al., 2021a; Aaser et al., 2022), and dairy cattle (Lomax et al., 2019; Verdon et al., 2020) are able to learn the paired stimuli of a VF system and accordingly respond to the AT only. This association was observed in sheep by day 3 and a mean of three paired stimuli (Marini et al., 2018a). Beef heifers have been found to require one (Verdon et al., 2021a) to six fence contacts (Campbell et al., 2018b) to learn to respond appropriately at the virtual boundary, with the highest learning curve within the first 2 d (Campbell et al., 2017) and up to 4 d in nonlactating dairy cows (Lomax et al., 2019). As a result of successful animal learning, the VF technology has been highly effective at keeping dairy cows within confined grazing areas (Lomax et al., 2019) or preventing beef cattle from entering a particular territory (Campbell et al., 2018a).

Many findings also suggest that cattle behavior and welfare are comparable between electric fencing (EF) and VF treatments, such as live weight, pasture utilization, herbage consumption, and fecal cortisol metabolites in beef heifers (Verdon et al., 2021a; Hamidi et al., 2022a), as well as milk production and cortisol concentrations, rumination and grazing time, activity behavior and total energy intakes in dairy cows (Langworthy et al., 2021; Verdon et al., 2021b). However, differences between EF and VF treatments have been reported in other studies; Hamidi et al. (2022b) found that heifers receiving an EP from a VF collar returned much faster to grazing activity than those receiving an EP from an EF, and Langworthy et al. (2021) found that daily pasture use by dairy cows was about 25% lower with VF than with EF.

To the best of our knowledge, the studies by Langworthy et al. (2021) and Verdon et al. (2021b) are unique in studying VF in lactating dairy cows, as research on VF has been initiated in beef or nonlactating cattle. However, lactating dairy cows are particularly sensitive to stress with a decrease in milk yield (von Keyserlingk et al., 2009). In addition, cortisol concentrations are readily available through milk analysis. Furthermore, dairy cows are kept at high stocking densities, require high forage quality to meet their needs, and therefore need to be moved more frequently in pasture-based systems (Langworthy et al., 2021). Therefore, research is needed to determine whether dairy cows can not only learn the concept of VF without compromising animal welfare but also transfer that knowledge to different paddocks. In addition, data on the longer-term effects of VF training on animal behavior and welfare are scarce, as highlighted by Colusso et al. (2020) and Verdon et al. (2021b).

Therefore, in the present study, we took advantage of the abovementioned benefits of using lactating dairy cows and investigated their learning progress and stress responses when introduced to a VF system over four consecutive experimental periods in which a new paddock was assigned each time. It was hypothesized that the cows would learn the concept of VF represented in a decreasing number of ATs and EPs over time and with each paddock change. Cow conditioning would be reflected in a decreasing EP/AT ratio until it eventually reaches zero. Consistent with the learning progress, we expected stress responses in the cows when introducing the VF system, which would reduce over time. Their stress response would be quantified by a significant change in the measured indicators compared to cows managed with EF, i.e.,

(1) Activity level, lying time, feed intake, body weight, and milk yield will either increase or decrease when activating the VF system, depending on the cow’s individual coping style as a response to the new situation (Van Reenen et al., 2005; Koolhaas and Van Reenen, 2016). Reactive coping is expressed by parasympathetic-induced behavioral inhibition (e.g., immobility, protection—withdrawal), whereas proactive coping involves a sympathetic-induced fight-or-flight response (Van Reenen et al., 2005). Accordingly, feed intake is expected to change as part of a behavioral or physiological response to stress with direct impacts on cow body weight and milk yield (Chen et al., 2015).

(2) Milk cortisol concentration will rise when activating the VF system and is higher in virtually fenced cows compared to those electrically fenced, as cortisol secretion is increased in response to stress (Boissy et al., 1998; Bristow and Holmes, 2007).

(3) The distance of the cows to the virtual boundary will change when activating the VF system, depending on whether the animals will approach or avoid the VF line based on individual personality traits (bold or shy).

(4) The occurrence of agonistic behaviors, vocalizations, and excretions will be more frequent when activating the VF system and is higher in virtually fenced cows compared to those electrically fenced. This assumption is based on previous studies showing increased aggression (Herskin et al., 2004; Polsky and von Keyserlingk, 2017), vocalization, and urination/defecation (Grandin, 1998; Rushen et al., 1999, 2001) as short-term indicators of discomfort in cattle exposed to stressors (e.g., slaughter, heat, separation, novelty, and fixation).

## Material and Methods

### Study area

The experiment was conducted during 59 consecutive days between August and October 2021 at the Agroscope research site in Posieux (FR), Switzerland (676 m above sea level, 46°46ʹN 7°06.5ʹE). According to the national weather service “MeteoSwiss,” there were 23 rainy days with total precipitation of 91.2 mm (mean ± SD: 1.5 ± 4.0 mm) during the experiment. Mean temperature was 14.4 ± 3.4 °C, ranging between 5.9 and 19.6 °C. All experimental procedures were approved by the Cantonal Veterinary Office of Fribourg according to the Swiss Animal Protection Ordinance (authorization number 2021-16-FR).

### Animals and housing

Forty-five lactating Holstein Friesian cows were housed in a ventilated free stall barn with cubicles and permanent access to a shaded concrete outdoor area. In the barn, the feed was supplied via weighing troughs (Insentec RIC System, Hokofarm Group, Emmeloord, the Netherlands) and concentrate feeding stations (Insentec RIC System, Hokofarm Group) using RFID. The diet consisted of a total mixed ration (**TMR**) of grass silage (20.8%), corn silage (37.9%), lucerne (21.6%), hay (17.1%), corn gluten (2.6%), and supplements of energy, protein, and minerals. The composition of the TMR was the same for all cows and was offered ad libitum. The supplemental feeds were provided to the animals individually per day and according to their needs. The cows were milked twice daily (0500 and 1600 hours) in a 5 × 4 tandem-milking parlor (Lemmer-Fullwood AG, Gunzwil, Switzerland) and were routinely weighed on a postmilking parlor scale (Insentec RIC System, Hokofarm Group).

### Experimental design

Of the 45 individuals, 20 were randomly selected and divided into four groups (2× VF treatment, **VF1** and **VF2**; 2× EF control, **EF1** and **EF2**) of five cows each for grazing. The groups were balanced according to lactation stage, which ranged from 121 to 326 d in milk (mean 238 d) in their second to seventh lactation (mean 3.9 lactations) at the beginning of the trial. All cows were accustomed to daily grazing with EF but had no experience with VF. At the beginning of the trial, all cows (*n* = 20) were fitted with a VF collar (Nofence AS, Batnfjordsør, Norway) and a pedometer on the right hind leg (IceQubes, Peacock Technology Ltd., Stirling, UK) by restraining them in a fixation stand. Both sensors were specifically designed for use in cattle. They remained on the animals throughout the experiment and were checked weekly for proper fit. The VF collars did not cause any adverse effects on the animals’ skin or coat. Wearing the pedometers caused slight chafing of the coat in two cows, so the pedometers were changed to the left hind leg. IceQube recordings have been validated in previous studies under different housing systems and attached to different legs, with moderate-to-strong agreement to visual observations or other monitoring devices (Elischer et al., 2013; Kok et al., 2015; Borchers et al., 2016; Charlton et al., 2022).

**Figure 1.**
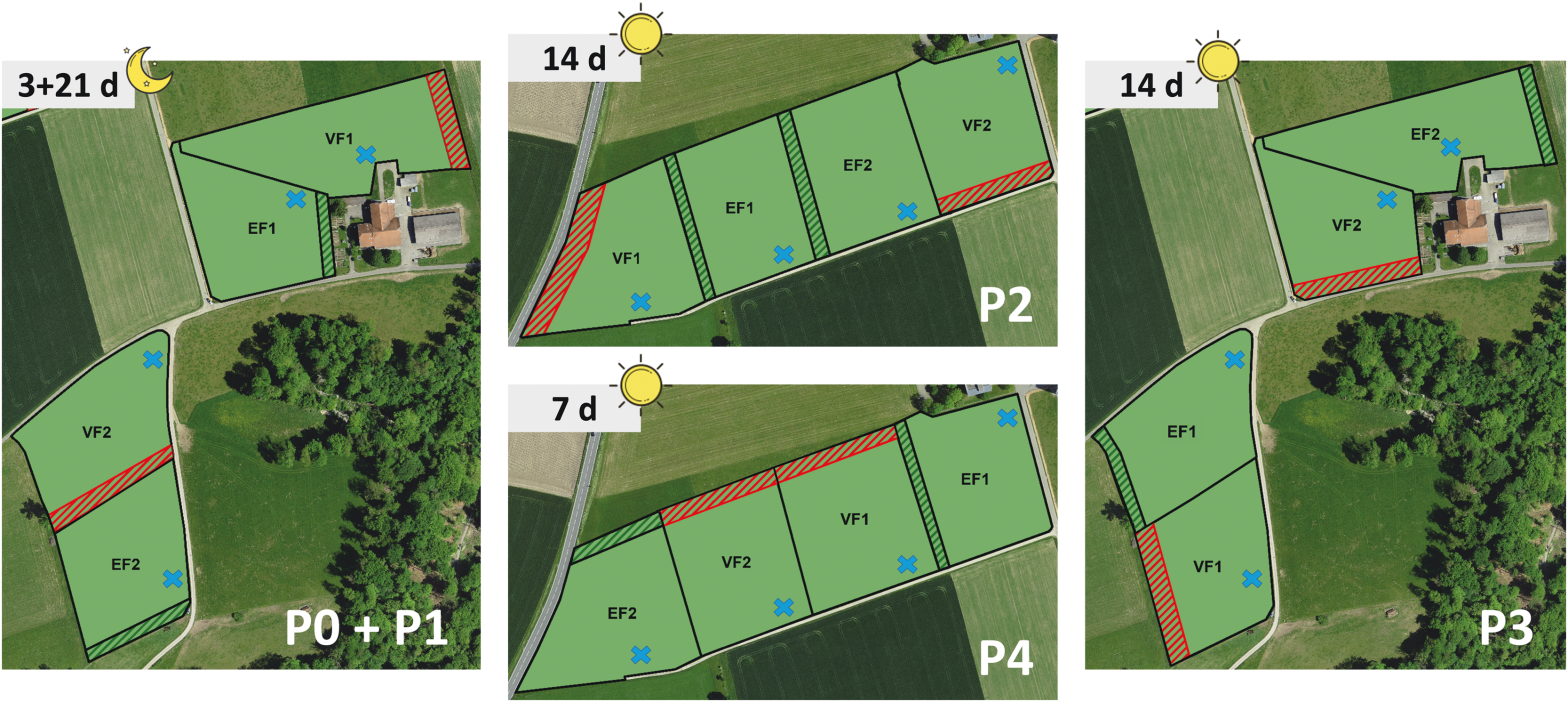
Overview of the paddocks during each experimental period (P1 to P4). In P1, each group (VF1, VF2, EF1, EF2) grazed in the paddocks for 21 half-days during nighttime (1700 to 0400 hours), in P2 and P3 for 14 half-days each, and in P4 for 7 half-days during daytime (0600 and 1500 hours). Each paddock was enclosed by an electric wire fence, marked with a solid black line. The inclusion zones in which the cows were allowed to stay are colored green (sizes in ha during P1/P2/P3/P4: **VF1** = 1.1/1.0/0.9/1.1, **VF2** = 1.0/1.0/0.9/1.0, **EF1** = 1.0/1.0/1.0/1.0, **EF2** = 1.0/1.1/1.1/1.1). The cross-hatched areas represent the exclusion zones of the control groups (in green) using an electric wire fence and of the treatment groups (in red) using VF (sizes in ha during P1/P2/P3/P4: **VF1** = 0.1/0.2/0.2/0.1, **VF2** = 0.1/0.1/0.2/0.1, **EF1** = 0.1/0.1/0.1/0.1, **EF2** = 0.1/0.1/0.1/0.1). The blue crosses represent the water troughs.

During the grazing period, each of the four groups grazed in a separate paddock for 3 half-days of lead-in period (**P0**), followed by four periods of experimental treatment (**P1 to P4**) of 21, 14, 14, and 7 half-days, respectively (Figure 1). During P0, the cows became accustomed to the experimental environment, their assigned group and wearing the sensors with deactivated virtual fence. In P1 to P4, the VF system was activated for both VF groups, with the duration of different periods adapted to the expected learning progress of the cows. Based on Campbell et al. (2017) and Lomax et al. (2019) showing that dairy cows were able to learn to respond to the AT only within a few days, it was expected that 3 wk during P1 in the present study would be sufficient for the cows to learn the association of the VF stimuli. As the experiment progressed, the duration of each period was shortened, as it could be assumed that the cows had already gained experience in VF. The grazing paddocks were electrically fenced, of comparable size of about 1 ha each, and with similar topography, vegetation structure, and botanical composition. In each enclosure, there was a water trough as indicated in Figure 1. To ensure comparable grazing conditions among periods, the grass was cut 2 wk before each paddock change. Within the paddocks of the VF groups, a virtual boundary was set parallel at a distance of about 10 to 15 m from the outer EF during P1 to P4. Similarly, a second EF was set parallel at a distance of about 10 to 15 m from one outer EF within the paddocks of the EF groups during P1 to P4 to allow comparability of paddock size and thus animal activity among all groups (Figure 1). During P0 and P1, cows grazed at night from about 1700 to 0400 hours to avoid heat stress, which is consistent with their natural preference during high temperatures (Hoy, 2009; Legrand et al., 2009). In P2 to P4, grazing was shifted to daytime from about 0600 to 1500 hours. For the other half of the days, the cows were indoors.

### Data collection

#### VF using Nofence

The Nofence system consists of a tracking collar for the animal and a smartphone app with a Geographic Information System. The app is used to define an “inclusion zone,” where the animals are allowed to stay or an “exclusion zone” to prevent the animals from entering a certain area. Once the animal approaches the virtual boundary, the VF collar emits an AT (82 dB at 1 m, 5 to 20 s depending on animal speed), followed by an EP (0.2 J for 1 s) at the lateral neck when the animal crosses it (Nofence AS, 2023). For comparison, electric fences commonly used in European cattle farming emit EPs of up to 5 J for <0.01 s upon fence contact (EN 60335-2-76:1999+A1:2001). The VF collars recorded the GNSS positions of each cow (*n* = 20) continuously throughout the experimental period of 59 d. The frequency of recording increased as an animal approached the virtual boundary, i.e., every 1/15 min at a distance <30 m, 1/s at 30 to 3.5 m, and 4/s at 3.5 to 0 m (Nofence AS, 2023). When the virtual boundaries were activated during P1 to P4, the VF collars recorded each AT and EP with a georeferenced time stamp and the warning duration of each AT in milliseconds. To minimize satellite positioning interference from surrounding factors, jammers (Nofence beacons) were installed throughout the barn. The beacons automatically deactivated the GNSS signal from the VF collars via Bluetooth or reactivated it as soon as the collars were outside a range of about 10 m.

#### Activity and lying behavior

Throughout the experiment, the IceQube pedometers continuously recorded cow movements in a 15-min interval based on a three-axis acceleration, which included lying time and a motion index (MI). The MI was automatically generated from the total leg acceleration and corresponded to a second-wise value between 0 (no movement) and 30 (strong movement; Peacock, 2010). Using the IceQubes fixed data sampling rate of 4 Hz and a reporting granularity of 15 min, MI was automatically accumulated over 900 s accordingly. Furthermore, IceQube pedometers automatically detected the onset of estrus events based on the movement pattern of each cow. This information was used to control for potential confounding effects of estrus during the experiment. The IceQubes recorded a total of 27 estrus events (0 in P0, 11 in P1, 4 in P2, 7 in P3, and 5 in P4) from 11 different cows (five EF control and six VF treatment) throughout the experiment. Of these, 14 estrus events (0 in P0, 5 in P1, 3 in P2, 4 in P3, and 2 in P4) from 8 different cows (four each in EF control and VF treatment groups) occurred on days of behavioral observation. Thus, the occurrence of estrus events was comparable between treatments and among experimental periods (as a percentage of the sample size). As a pre-analysis revealed no confounding effects on the observed behaviors, the data was not included in further analysis.

#### Behavioral observations

Stress-related behavioral responses were directly observed during the first 2 h of grazing after milking, as we expected the cows to be more active on pasture during this period as indicated in previous studies (Gibb et al., 2008; Ueda et al., 2011). Accordingly, behavior was monitored from about 1700 to 1900 hours during P0 and P1 and from about 0600 to 0800 hours during P2 to P4. The observations were conducted daily during P0 and on days 1, 2, 3, 7, 14, and 21 of P1, on days 1, 2, 3, 7, and 14 of P2 and P3, and on days 1, 2, 3, and 7 of P4. In determining the days of observation, we assumed that fence contact would be most frequent at the beginning of each period, respectively, in a new paddock, and would decrease towards the end. The observations were carried out from a raised platform (approximately 2.5 m high) by two observers at the same time, each monitoring one EF and one VF group in parallel (*n* = 10 cows per observer). If necessary, binoculars were used to ensure the correct identification of individual cows. Observer 1 was always the same person, observer 2 alternated between two people. All observers were instructed at the beginning of the experiment in a 2-h training session to directly observe the cows’ reaction after receiving an AT and/or EP at the virtual boundary or an EP at an electric fence, the frequency of elimination behaviors (urination and defecation) and occurrence of agonistic interactions (chase and displacement) (for the definition of behaviors, see Table 1). Interobserver variability between observer 1 and observer 2 alternates during training was *k* = 0.71 and 0.56 (*P* < 0.05), respectively, representing fair to good strength of agreement according to the classification of Fleiss et al. (2003). To identify the individuals on pasture, the cows were marked with numbers using a yellow marking spray. The marking was refreshed before each behavioral observation. The behaviors were recorded as point events using the open-source software “BORIS” (Version 7.12.2) for Microsoft Windows (Friard and Gamba, 2016).

**Table 1.** Ethogram of behaviors that were recorded in the pasture on each of the 23 observation days during the experiment for the EF and VF groups

Behavior	Definition
Elimination	Urination or defecation, regardless of contact with the virtual or electric fence
Vocalization	Any type of vocalization. Vocalizations are recorded for each individual call, regardless of contact with the virtual or electric fence
Retreat VF	After contact with the virtual fence (receiving AT and/or EP), the cow turns around and walks away for at least 1 body length. The cow stays inside the inclusion zone of the VF paddock (i.e., it does not enter the exclusion zone)
Retreat EF	After contact with an electric fence (receiving an EP) in the VF or EF paddock, the cow turns around and walks away for at least 1 body length. The cow stays inside the inclusion zone (i.e., it does not enter the exclusion zone)
Run VF	After contact with the virtual fence (receiving AT and/or EP), the cow turns around and runs away at trot or canter for at least 1 body length. The cow stays inside the inclusion zone of the VF paddock (i.e., it does not enter the exclusion zone)
Run EF	After contact with an electric fence (receiving an EP) in the VF or EF paddock, the cow turns around and runs away at trot or canter for at least 1 body length. The cow stays inside the inclusion zone (i.e., it does not enter the exclusion zone)
Escape VF	A cow crosses the virtual fence and enters the predefined exclusion zone at any speed (walk, trot, gallop). After the escape, the cow may remain within the exclusion zone or immediately return to the inclusion zone of the VF paddock
Escape EF	A cow crosses an electric fence of the VF or EF paddock at any speed (walk, trot, gallop). After the escape, the cow may remain outside its predefined paddock or immediately return to its inclusion zone
Bucking VF	After contact with the virtual fence (receiving AT and/or EP), both or at least one hind leg lifts off the ground and is kicked backwards at any speed (walk, trot or canter)
Bucking EF	After contact with an electric fence (receiving AT and/or EP) in the VF or EF paddock, both or at least one hind leg lifts off the ground and is kicked backwards at any speed (walk, trot, or canter)
Chase	Cow runs after another cow in trot or gallop over a distance of at least 2 body lengths. The behavior is recorded for the chasing animal, not for the chased
Displacement	Cow pushes another cow by physical contact with its head or body. The behavior is recorded for the displacing animal, not the displaced

All behaviors listed were recorded as point events.

#### Feed intake indoors

To determine whether the use of the VF system influenced the feeding behavior of the cows, feed intake was measured during the daily intergrazing periods when the cows remained indoors, i.e., between approximately 0500 and 1700 hours during P0 and P1, and between approximately 1600 and 0600 hours during P2 to P4 (milking time included). This allowed data on an individual basis, which was not available in the pasture. Thus, individual intakes of TMR and supplemental feeds, both in kilogram fresh matter per day, were recorded separately by the feeding system.

#### Milk production and body weight

Individual milk yield (kg) and body weight (kg) were routinely recorded at each milking. Milk fat (%) and protein (%) content per cow were measured every 2 wk as part of the regular milk performance testing, which took place a total of four times during the trial. All data recorded were accessed via the herd management system after the experiment.

#### Milk sampling and cortisol analysis

Milk samples were collected during the regular milking times of the farm. During P0, samples were taken twice per day to obtain reference levels of milk cortisol and its diurnal variation in the morning and evening milking. During P1 to P4, milk samples were taken always after grazing on days 1, 2, 3, 7, 14, and 21, depending on the length of each period, which corresponded to the sampling interval for the behavioral observations. In P1, milk samples were collected during morning milking and in P2 to P4 during evening milking. For milk sampling, an extract of total milk was collected from each cow in a bottle using a milk sampler (Pulsameter 2, Lemmer-Fullwood AG) according to the standard practice for milk performance testing. After manual mixing of the sample, about 5 mL of it was individually filled into plastic tubes (Sarstedt AG & Co. KG, Numbrecht, Germany) and frozen at −18 °C until laboratory analysis. During the entire experimental period of 59 d, only one sample from one cow was missing, so a total of 467 milk samples were obtained. The laboratory analysis of milk cortisol was performed in milk serum by using a salivary cortisol ELISA kit (Salimetrics LLC., State College, PA, USA). After thawing, whole milk was skimmed by centrifugation at 3,000 × *g* for 15 min. The supernatant was centrifuged again at 14,000 × *g* for 30 min to obtain milk serum. Analyses were performed in duplicate. The detection limit of the assay was 0.1 ng/mL. The intra- and inter-assay CV were 7.3% and 5.6%, respectively. Before use, the assay was validated for its suitability in pooled milk by spiking samples with cortisol assay standard at concentrations throughout the detection range of the assay. The calculated recoveries of the spiked samples ranged from 96% to 105%.

#### Grass measurement

Grass height was measured at the beginning and end of P0 to P4 on each paddock using an electronic rising plate meter (**RPM**; Model EC10, Jenquip, Feilding, NZ). A total of 250 RPM measurements were carried out on each paddock, consisting of 200 RPM drops from two measurement repetitions in the inclusion zone and 50 RPM drops from one measurement in the exclusion zone. During sampling, both zones were walked in a W-shape. Grass height measurements were only used to monitor forage availability in the paddocks during the experiment. The data (Supplementary 1) were not used for further analysis.

### Data processing

Of the 20 cows used in the experiment, data from two cows had to be excluded due to disease unrelated to the study. Thus, data from 18 cows were analyzed, of which 10 were into the EF groups and 8 were into the VF groups.

#### Nofence stimuli

The recordings of ATs and EPs were used to examine the learning progress of the cows. For this purpose, we calculated the weekly ratio of EP/AT. The smaller the ratio, the more ATs relative to EPs were received by a cow, indicating an improvement in the cow’s awareness of the AT. For model fitting, data were prepared by determining ATs and EPs on a daily basis for each cow of the VF groups according to the time stamps. The recorded warning duration in milliseconds was added up per day and converted into seconds.

#### Relative distance from the exclusion zones

To determine the effect of VF on the spatial distribution of animals near the virtually or physically excluded zone in each paddock, we analyzed the GNSS positions recorded by the VF collars. The positions were projected into the Swiss national grid CH1903+ LV95 and all positions outside a 5-m buffer around the paddocks were deleted. For each position, we calculated the shortest linear distance to the virtual or physical exclusion zone. Because not all paddocks had identical shapes, distances were expressed as relative distances by dividing them by the maximum distance from the virtual or physical exclusion zone. Daily averages of relative distances were calculated for each individual.

#### Activity and lying behavior

For MI and lying time, we identified 12 and 30 missing 15-min values for two pedometers, resulting in a reduced data size for the behaviors of one EF cow and one VF cow, respectively. We calculated MI and lying time on a daily basis per cow by summing up their total number of 15-min values per day.

#### Behavioral observations

During the observations, a total of eight behaviors were recorded for the VF and EF groups, i.e., elimination, vocalization, chase, displacement, retreat VF or EF, running VF or EF, escape VF or EF, and bucking VF or EF. Interactions with the electric fence were not observed throughout the experiment, so no “retreat EF,” “running EF,” “escape EF,” and “bucking EF” were recorded. Thus, the analysis of these behaviors concerns the VF groups only. Bucking was recorded only five times during the entire experiment and only by a single cow after receiving one EP at the virtual boundary on one day during P1. Therefore, due to its low frequency, it was removed from the analysis. Moreover, the records of one observer were lost for a single day (D1 of P2) due to a technical defect of a laptop. Because the missing record affected one EF and one VF group equally, we kept the corresponding observation day within the dataset to support the sample size for analysis. The missing values were replaced by the individual mean value per cow at the beginning of the periods for each behavior.

#### Milk production, body weight, and feed intake

For milk yield and body weight, mean values per cow and day were calculated. Based on the milk performance tests, we derived the mean and SD of milk fat and protein from four values per cow. To account for the total amount of individual feed intake indoors per day and to simplify its inclusion for further analysis, we combined TMR and supplementary feeds to the output variable “net lactation energy” (**NEL**) per cow and day. Daily NEL intake per cow (in MJ) was calculated from the energetic values of the dry matter content of each TMR and supplement component multiplied by the daily amount of fresh feed consumed per cow.

### Statistical analysis

Linear (**LMER**) and generalized (**GLMM**) mixed-effect models were fitted to test whether target variables (Tables 2 and 3) depended on groups, periods, and time within periods (**TWP**). The reason for including TWP was that the cows were moved to a new paddock at the beginning of each period and the treatment groups had to adapt to a new virtual boundary. We hypothesized that the learning process, corresponding to the temporal progression within periods, leads to a change in the target variable. This would be reflected in either an increased or decreased target variable value at the beginning of each period, which would then significantly change over time. Therefore, TWP was used as a categorical variable with the three factors “beginning” (**B**), “middle” (**M**), and “end” (**E**) of each period, depending on their respective durations. For B, all data obtained on day 1 of P0, days 1 to 7 of P1, days 1 to 5 of P2 and P3, and days 1 to 2 of P4 were considered. For M, data collected on days 2 of P0, days 8 to 14 of P1, days 6 to 9 of P2 and P3, and days 3 to 5 of P4 were considered. For E, data obtained on day 3 of P0, days 15 to 21 of P1, 10 to 14 of P2 and P3, and days 6 to 7 of P4 were considered. Since behavioral observations and milk sampling for determining cortisol concentrations were not conducted daily during the experiment, but on specific days at the beginning, middle, and end of each period (Figure 1), their measurements were also categorized according to TWP for further analyses.

**Table 2.** Results of the fitted linear mixed effects models (LMER) for the parameters of interest in evaluating dairy cow welfare and productivity observed on a total of 18 cows (*n* = 10 control; *n* = 8 VF treatment)

Target variable	Transformation	*n* (*N*_days_ × *N*_animals_)	Mean^1^	Standard deviation^1^	Data range^1^	Random effects	Fixed and interaction effects			
							Variables	Bootstrap test-statistic	*P*-value	Significance level^2^
NEL intake, MJ	No	(60 × 18)^3^	69.1	20.9	134.2	Cow nested in Group Day	EF vs. VF groups Period TWP Period × TWP Period × EF vs. VF groups	1.33 50.46 7.77 46.21 20.80	0.312 <0.001 0.031 <0.001 0.001	ns *** * *** **
Body weight, kg	No	(60 × 18)^3^	704	37	209	Cow nested in Group Day	EF vs. VF groups Period TWP Period × TWP	0.81 62.47 13.74 31.33	0.431 <0.001 0.003 0.004	ns *** ** **
Milk yield, kg	No	(60 × 18)^3^	26.7	6.3	37.0	Cow nested in Group Day	EF vs. VF groups Period TWP Period × EF vs. VF groups	0.528 61.36 9.50 19.55	0.534 <0.001 0.018 0.002	ns *** * **
Milk cortisol, ng/mL	Log()	(26 samples × 18) -1 sample	0.73	0.45	2.59	Cow nested in Group Day of sampling Time of sampling	EF vs. VF groups Period TWP	0.03 26.83 11.86	0.899 <0.001 0.007	ns ** **
Motion index	Log()	(60 × 18)^3^	8,985	3,254	36,966	Cow nested in Group Day	EF vs. VF groups Period TWP Period × EF vs. VF groups	5.23 10.84 11.74 11.85	0.228 0.064 0.021 0.018	ns ns * *
Lying duration, min	No	(60 × 18)^3^	579	127	820	Cow nested in Group Day	EF vs. VF groups Period TWP Period × EF vs. VF groups	0.20 15.40 17.84 26.61	0.694 0.016 0.001 <0.001	ns * ** ***
Relative distance, %	No	(60 × 18)^3^	0.41	0.09	0.64	Cow nested in Group Day	EF vs. VF groups Period TWP Period × EF vs. VF groups	0.04 73.52 12.66 49.28	0.839 <0.001 0.005 <0.001	ns *** ** ***

The target variables were recorded on a total of 18 cows (*n* = 10 control; *n* = 8 VF treatment) throughout 59 d (except milk cortisol, which was recorded on 26 d).

^1^Before transformation.

^2^Significance levels indicated by *P* < 0.001 (***), *P* < 0.01 (**), *P* ≤ 0.05 (*), and *P* > 0.05 (ns).

^3^60 instead of 59 d due to double count of one date when changing from night to daytime grazing.

TWP, time within periods; EF, electrically fenced groups; VF, virtually fenced groups.

**Table 3.** Results of the fitted generalized mixed effects models (GLMM) using the Template Model Builder (glmmTMB) to evaluate dairy cow behavior observed on a total of 18 cows (*n* = 10 control; *n* = 8 VF treatment) over 23 d as well as the VF recordings of 8 cows within 56 d (= 8 wk)

Target variable	Model family	*n* (*N*_days_ × *N*_animals_)	Mean	Standard deviation	Data range	Random effects	Fixed and interaction effects				
							Variables	χ2 test-statistic	Df	*P*-value	Significance level^1^
Audio tones, AT count	glmmTMB (nbinom)	(57 × 8)^2^	1.89	3.30	26.0	Cow nested in Group Day	Period TWP TWP × Period	12.19 18.10 21.70	3 2 6	0.007 <0.001 0.001	** *** **
Electric pulses, EP count	glmmTMB (zi, poisson)	(57 × 8)^2^	0.12	0.65	8.0	Cow nested in Group Day	Period TWP	15.13 11.80	3 2	0.002 0.003	** **
Warning Duration, s	glmmTMB (zi, nbinom)	(57 × 8)^2^	11.55	37.78	428.4	Cow nested in Group Day	Period TWP TWP × Period	3.03 8.04 33.46	3 2 6	0.386 0.018 <0.001	ns * ***
Displacement, count	glmmTMB (nbinom)	(23 × 18)	0.35	0.95	10.0	Cow nested in Group Day of observation Time of observation	EF vs. VF groups Period TWP	3.86 5.23 0.56	1 4 2	0.050 0.182 0.755	* ns ns
Chase, count	glmmTMB (nbinom)	(23 × 18)	0.05	0.39	6.0	Cow nested in Group Day of observation Time of observation	EF vs. VF groups Period TWP	1.53 6.26 0.28	1 4 2	0.216 0.181 0.869	ns ns ns
Elimination, count	glmmTMB (poisson)	(23 × 18)	1.10	0.87	4.0	Cow nested in Group Day of observation Time of observation	EF vs. VF groups Period TWP	0.05 3.76 0.23	1 4 2	0.821 0.440 0.889	ns ns ns
Vocalization, count	glmmTMB (nbinom)	(23 × 18)	0.37	1.59	22.0	Cow nested in Group Day of observation Time of observation	EF vs. VF groups Period TWP TWP × Period	3.53 2.68 0.55 25.20	1 4 2 8	0.060 0.612 0.873 0.002	ns ns ns **
Retreat, count	glmmTMB (poisson)	(20 × 8)	0.09	0.39	3.0	Cow nested in Group Day of observation Time of observation	Period TWP	2.81 7.60	3 2	0.423 0.022	ns *
Run, count	glmmTMB (quasi-Poisson)	(20 × 8)	0.07	0.60	10.0	Cow nested in Group Day of observation Time of observation	Period TWP	10.06 1.56	3 2	0.018 0.459	* ns

^1^Significance levels indicated by *P* < 0.001 (***), *P* < 0.01 (**), *P* ≤ 0.05 (*), and *P* > 0.05 (ns).

^2^57 instead of 56 d due to double count of one date when changing from night to daytime grazing.

nbinom, negative binomial; zi, zero-inflated; TWP, time within periods; EF, electrically fenced groups; VF, virtually fenced groups; Df, degrees of freedom.

All statistical analyses and generation of figures were carried out in R Version 4.2.2. LMER and GLMM were fitted depending on the distribution of the target variables, which were previously checked using histograms, QQ plots, and boxplots. This also provided information about any outlier in the dataset. The LMER were computed using the R package “lme4” (Bates et al., 2014). Milk cortisol and MI were log-transformed to meet model requirements of Gaussian distribution and homoscedasticity. For the other target variables, no transformation was needed. The LMER were fitted with group, period, and TWP as fixed effects, and day and cow nested in group as random effects (Table 2). For the analysis of milk cortisol, the time of milk sampling was additionally added as a random effect. The final models were selected by backward elimination based on a significant *P*-value in bootstrap testing using the R package “pbkrtest” (Halekoh and Højsgaard, 2021). Finally, pairwise post-hoc comparisons were performed for the significant main and interaction effects using the R package “emmeans” (Lenth, 2022).

For fitting GLMM, the Template Model Builder, package “glmmTMB” (Brooks et al., 2017), was used. The GLMM were fitted with period and TWP as fixed effects, and day and cow nested in group as random effects (Table 3). To analyze the individual cow effect on the number of AT, EP, and warning duration, cow was tested for significance as a random factor using a likelihood ratio test (**LRT**) with the function “lrt().” For the analyses of cow behaviors (displacement, chase, elimination, and vocalization), group, period, and TWP were considered as fixed effects as well as day, cow nested in group and the time of observation as random effects (Table 3). The function “descdist” of the R package “fitdistrplus” (Delignette-Muller and Dutang, 2015) was used to bootstrap the target variables of animal behaviors on a skewness-kurtosis diagram to identify the best distribution(s) to fit the models. Based on this, several likelihood structures were considered for analysis, including negative binomial, Poisson, and quasi-Poisson, with and without zero inflation. The models were compared using AIC, BIC, and the *P*-values of the chi-square test statistics obtained from LRTs with the “anova()” function. Based on the statistical criteria, the final models were determined. Finally, each of the fitted model (LMER and GLMM) was tested with the R package “DHARMa” (Hartig, 2022) for goodness of fit of the simulated residuals, including tests for distribution, over-/under dispersion, outliers, and zero inflation.

**Figure 2.**
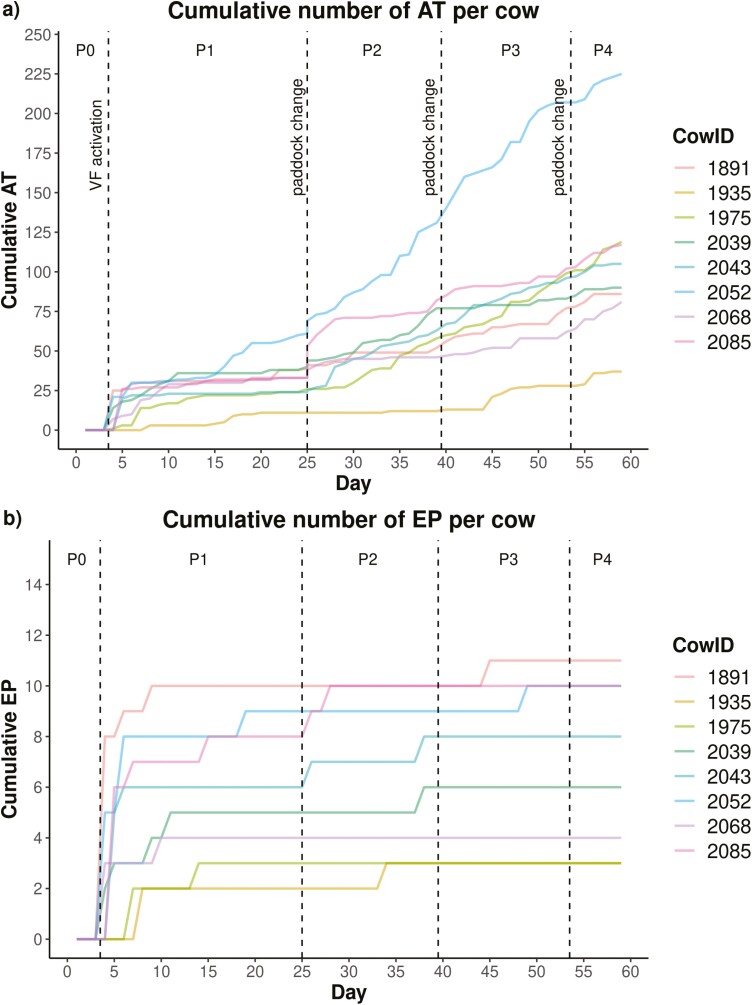
Cumulative number of (a) ATs and (b) EPs received by each cow during the lead-in period (P0) and periods 1 to 4 (P1 to P4). The vertical dashed lines in gray represent the beginning of an experimental period, when the cows were moved to a different paddock with a new virtual boundary.

**Figure 3.**
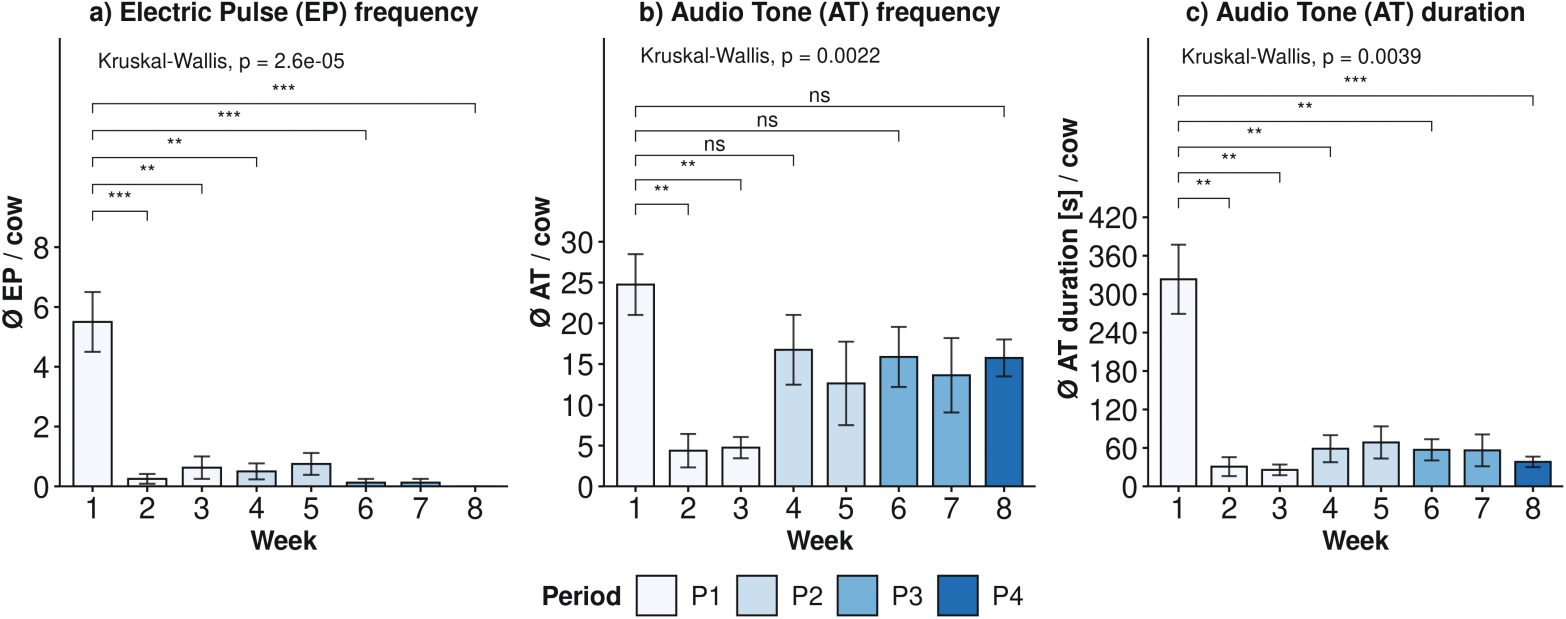
Mean number of (a) EPs per cow, (b) ATs per cow, and (c) mean warning duration per cow (in s) of eight animals during each week of the experiment. Error bars indicate the standard error. As cows were moved to a new paddock at the beginning of each period (Weeks 4, 6, and 8), differences from Week 1 were determined using the Kruskal–Wallis test. Also, differences between Week 1 and Weeks 2 and 3 were tested, as P1 represented the key period of learning. Differences are indicated by significance levels of *P* < 0.001 (***), *P* < 0.01 (**), *P* ≤ 0.05 (*), and *P* > 0.05 (ns).

**Figure 4.**
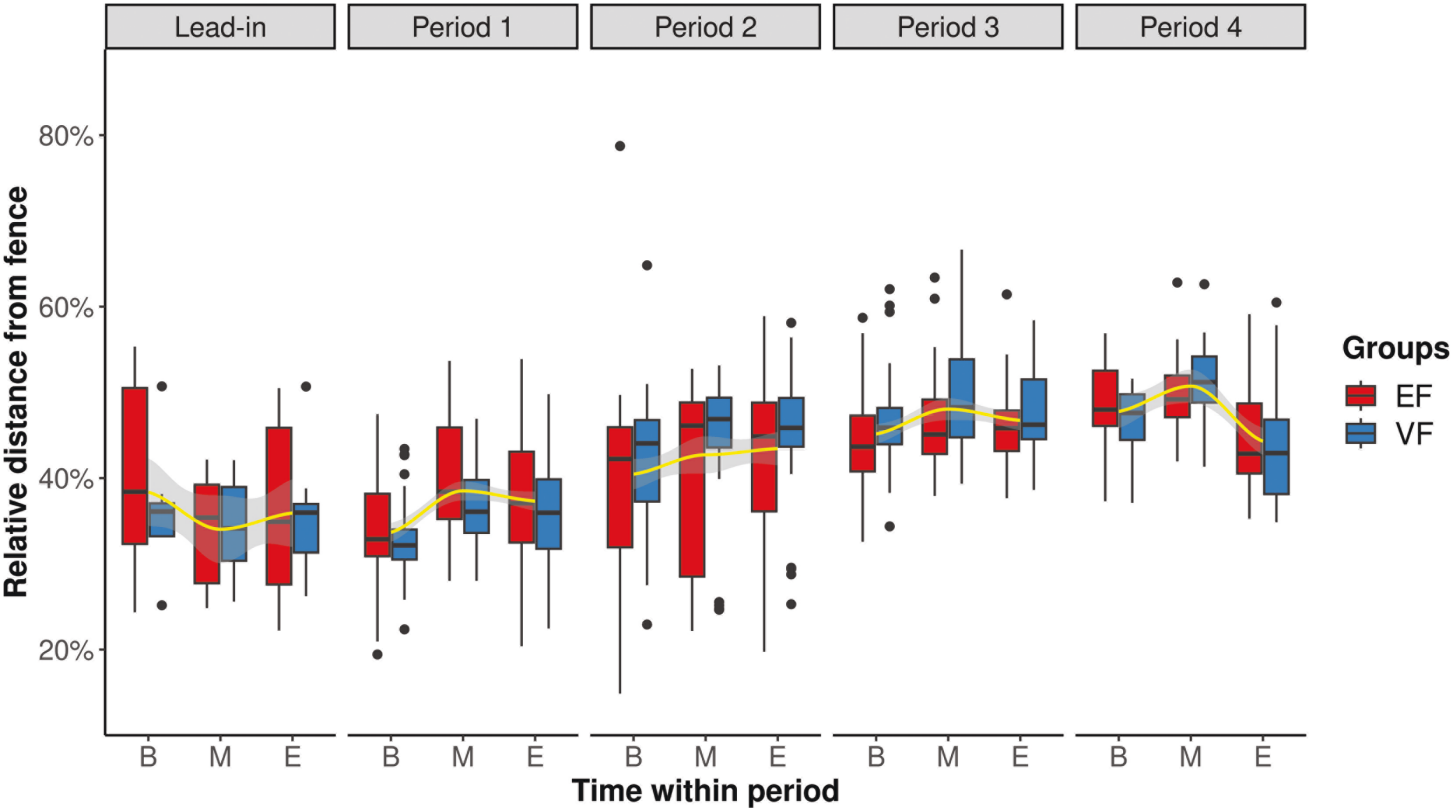
Relative distance from 0% to 100% of EF (*n* = 10) and VF (*n* = 8) groups from their exclusion zones within each paddock during the lead-in period (P0) and periods 1 to 4 (P1 to P4). The yellow line indicates the mean of all cows (*n* = 18) at the beginning (B), middle (M), and end (E) of each experimental period, along with the corresponding 95% CI within the gray shading.

**Figure 5.**
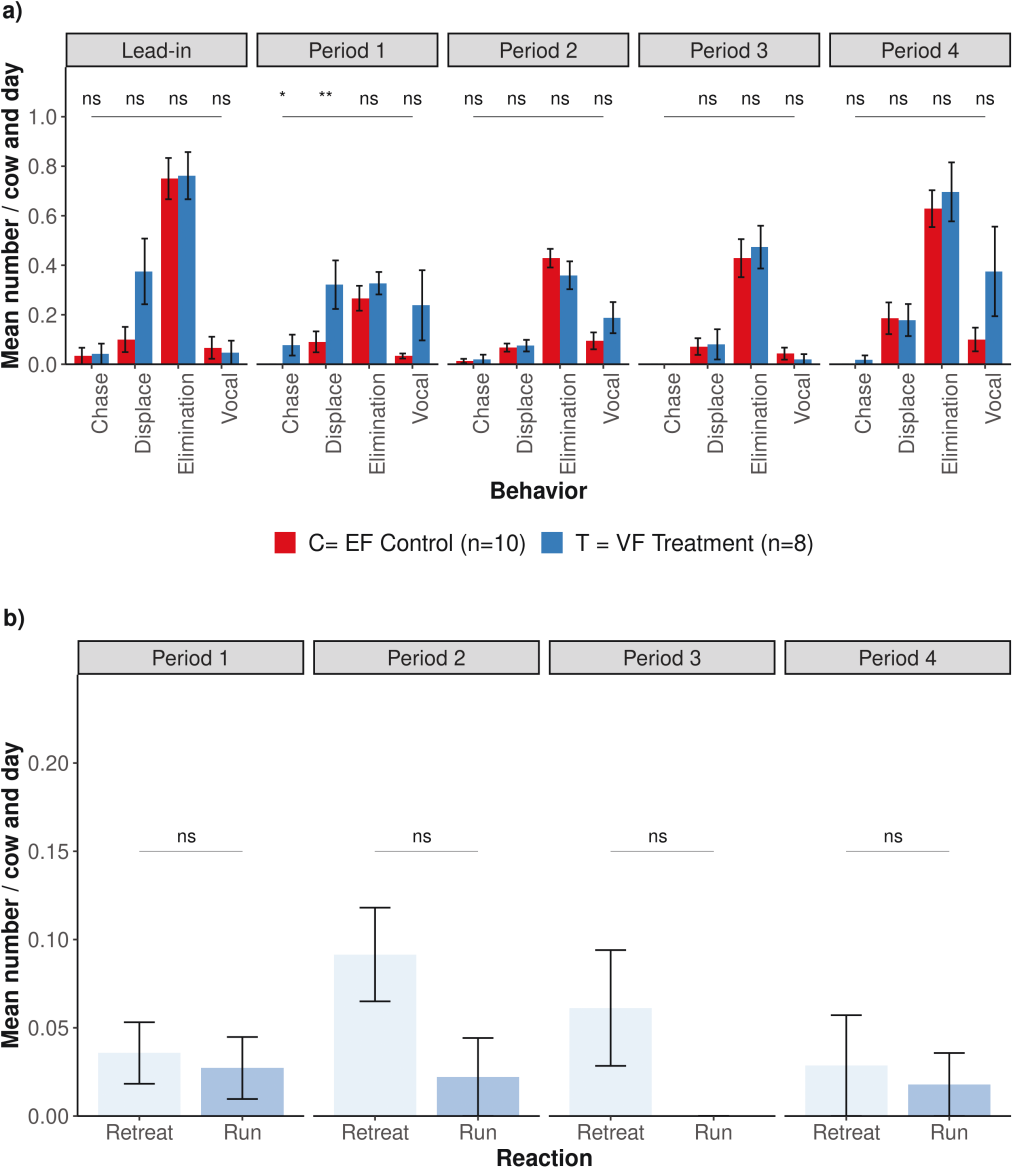
Mean number of (a) observed behaviors per cow and observation day across EF control (*n* = 10) and VF treatment (*n* = 8) groups during each experimental period, and (b) observed reactions at the virtual fence per cow and observation day across the VF treatment (*n* = 8) by period of an activated VF system (P1 to P4). There were 3 observation days during P0, 6 observation days during P1, 5 observation days each during P2 and P3, and 4 observation days during P4. Error bars indicate SE. Differences between EF and VF groups are indicated by significance levels *P* < 0.01 (**), *P* ≤ 0.05 (*), and *P* > 0.05 (ns), respectively, based on the *P*-value calculated by Wilcoxon rank-sum test.

**Figure 6.**
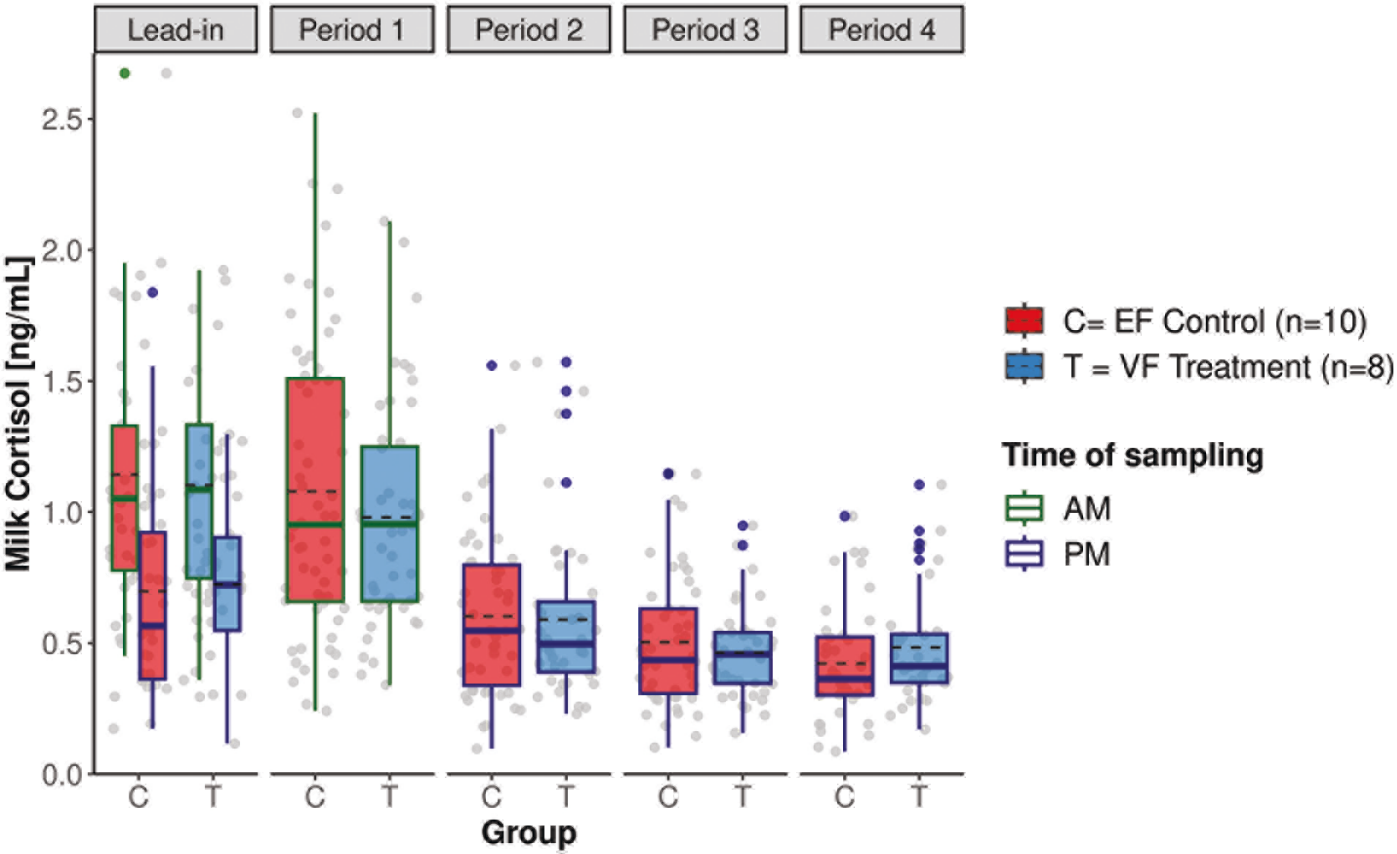
Milk cortisol concentrations of the EF control (*n* = 10) and VF treatment (*n* = 8) groups analyzed from 26 milk samples during lead-in (P0) and periods 1 to 4 (P1 to P4). During P0, milk samples were collected twice daily, i.e., during morning and evening milking. During P1 to P4, milk samples were taken always after grazing, i.e., during morning milking in P1 and during evening milking in P2 to P4. The plotted values refer to the raw data of milk cortisol (before log transformation), with the median shown as solid and the mean as dashed black line.

The figures were created using the following R packages: “ggplot” (Ginestet, 2011) for Figure 2 (design adopted from Aaser et al. (2022)), Figures 3–6; “ggpubr” (Kassambara, 2023) for Figures 3 and 5.

## Results

### Nofence stimuli

Throughout the experiment, the VF system successfully kept all cows in their assigned inclusion zones. No escape, i.e., crossing the virtual boundary and receiving three successive paired stimuli, was recorded. The cows received 860 ATs and 55 EPs throughout 56 d of activated virtual boundary. The total number of stimuli per cow ranged from 37 to 225 ATs (mean ± SD: 1.9 ± 3.3 AT per day) and 3 to 11 EPs (mean ± SD: 0.1 ± 0.7 EP per day), indicating differences among individuals. Indeed, cow as a random factor had a significant effect on the number of ATs (χ^2^ = 25.6, *P* < 0.001) and on the mean warning duration (χ^2^ (1, *N* = 456) = 7.18, *P* < 0.001), while it was similar for the number of EPs (χ^2^ = 0.22, *P* = 0.638). Overall, it took a mean (±SD) of 7.9 ± 4.0 paired stimuli (AT followed by an EP) at the virtual boundary until the cows responded to the AT only.

As shown in Figure 2, the maximum number of EPs per day for a single cow was eight and occurred once on day 1 when introducing the animals to VF. At herd level, the total number of EPs reached a peak of 18 EPs on day 1, but it decreased to 5 EPs on day 3 and remained below that threshold for the remaining part of the experiment. Thus, the animals received 64% of all EPs during the first three half-days of VF grazing, which was on average 1.5 times higher per cow and day than during the rest of the adaption period. As shown in Figure 2, the frequency of ATs increased again on day 25, when the cows were moved to a new paddock. However, this increase was less intense than at the beginning of the trial. The mean number of ATs per cow far exceeded that of EPs during each week, with EPs decreasing significantly (*P* < 0.001) over time (Figure 3). In P3, only two EPs were triggered and not at the beginning and in P4 (respectively week 8), no EP was received at all (Figure 3). The mean ratio of EP/AT decreased from week 1 (mean ± SD: 0.22 ± 0.07) to week 4 (mean ± SD: 0.02 ± 0.05), week 6 (mean ± SD: 0.01 ± 0.02), and week 8 (mean ± SD: 0.00 ± 0.00). Furthermore, the mean duration of warnings per cow significantly (*P* < 0.001) decreased over time, while the number of ATs during week 1 was comparable to weeks 4 to 8 (Figure 3). A dip of ATs and a low mean warning duration can be noted in weeks 2 and 3 (Figure 3). The significant effect of period and TWP, as well as their interaction effect was also evident in the GLMM for the number of ATs, EPs, and warning duration (Table 3).

### Spatial use of pastures

Throughout the experimental period of 59 d, the relative distance from the exclusion zone within each paddock did not significantly differ between EF (mean ± SD: 42 ± 1.3%) and VF groups (mean ± SD: 42 ± 1.4%). However, as indicated in Table 2, we found changes among periods (*P* < 0.001) and in TWP (*P* = 0.005). In P0 and P1, the relative distance of the EF and VF groups to the exclusion zone was smaller, each with a mean of 36%, compared to P2 (42%), P3 (47%), and P4 (47%; Figure 4). In addition, the distance at the beginning of each period was on average 3.1% lower than at the middle (*P* < 0.01) and 2.2% lower than at the end (*P* < 0.05) of each period. In P1 and P2, the variation in distance of the EF group was larger compared with the VF group (Figure 4). A closer look at the groups revealed that in P1 it was mainly EF1 and in P2 mainly EF2 that caused the large variation within the EF treatment. In Figure 4, the TWP categories of P1 correspond to the experimental weeks in Figure 3. Week 1 (B) shows a high number of ATs per cow compared to weeks 2 and 3 and correspondingly, cows stayed closer to the virtual boundary in week 1 compared to weeks 2 and 3. In P2 to P4, the number of ATs is back at a higher level, despite keeping a higher distance to the virtual boundary in these periods.

### Activity and lying behavior

A mean (±SD) for MI of 8,985 ± 3,254 per day and a mean (±SD) for lying time of 579 ± 127 min per day was measured. Based on LMER, MI and lying time did not differ significantly between EF and VF groups (Table 2). The LMER revealed that MI was similar among periods, but differed in TWP (*P* = 0.021), with scores being 763 lower at the end of each period than at the beginning (*P* < 0.05). Lying time differed among periods (*P* = 0.016) and in TWP (*P* = 0.001), Table 2. A post-hoc test indicated that mean lying time decreased by 73 min per day during P1 than during P2 (*P* < 0.01) and decreased by 84 min per day on average at the end of each period compared to its beginning (*P* < 0.01).

### Behavioral observations

Throughout the 23 d of observation, we recorded 151 (mean ± SD: 8.5 ± 11.3 per cow) vocalizations, 458 (mean ± SD: 25.4 ± 8.7 per cow) elimination behaviors, 145 (mean ± SD: 8.1 ± 6.2 per cow) displacements, and 19 (mean ± SD: 1.1 ± 2.2 per cow) chases. The frequency of the observed behaviors did not differ significantly among periods and in TWP (Table 3).

In P1, agonistic behaviors were significantly higher (*P* < 0.01 for displacement and *P* < 0.05 for chase) in the VF groups than in the EF groups (Figure 5a). There were on average 5.9 more displacements observed in the VF groups (*P* < 0.05) than in the EF groups with a significant effect in P1. However, as shown in Figure 5a, displacements were also observed to a greater extent for the VF groups during P0, but without significant effect. The number of observed eliminations was similar for the EF and VF groups in all experimental periods. As shown in Figure 5a, vocalizations visually have the strongest effect in P4, although no EP were recorded in this period.

In the reaction of the VF groups at the virtual boundary, there were 39 (mean ± SD: 4.9 ± 4.3 per cow) retreats and 29 (mean ± SD: 3.6 ± 4.9 per cow) runs throughout the 23 observation days. There is an indication that the responses of the cows changed over the course of the experiment (Figure 5b). Although the findings were not significant, they show the same pattern as the EP in Figures 2 and 3. Running seemed mainly present in P1, whereas cows increasingly retreated in P2 and P3.

### Feed intake

The cows had a mean (±SD) intake of 20 ± 6 kg TMR fresh matter per day and 1.8 ± 1.8 kg supplements fresh matter per day, resulting in a mean (±SD) NEL intake of 69 ± 21 MJ per cow and day in the barn. Although, we found differences among periods (*P* < 0.001) and in TWP (*P* = 0.031), the EF and VF groups did not differ significantly in mean NEL intake (Table 2). NEL intake was higher during P3 and P4 with a mean (±SD) of 84 ± 3.4 and 78 ± 3.8 MJ per cow and day, respectively, compared to P0 at 73 ± 4.7 and P1 at 60.4 ± 3.3 MJ per cow and day. Within each period, NEL intake was on average 2 MJ per cow and a day higher by the end of each period compared to the beginning (*P* < 0.05). Therefore, no negative pattern on mean NEL intake was identified across the four experimental periods.

### Body weight

The mean (±SD) body weight was 704 ± 37 kg per cow, ranging from 611 to 819 kg per cow. The LMER indicated no significant differences between EF and VF groups (Table 2), but among periods (*P* < 0.001) and in TWP (*P* = 0.003). Body weight was the lowest in P0 with a mean of 697 kg per cow and increased with each additional period until finally reaching the highest mean weight of 717 kg per cow in P4. Within a period, body weight decreased by an average of 3.4 kg per cow at the end of each period compared to the beginning (*P* < 0.01).

### Milk production

Throughout the experiment, mean (±SD) milk yield was 26.7 ± 6.3 kg per day, fat content was 4.3 ± 0.5% per day, and protein content was 3.6 ± 0.2% per day. The cows showed a daily variation in milk production, with mean (±SD) yield being 2.4 ± 0.7 kg higher in the morning (*P* < 0.001) than in the evening milkings. Again, mean milk yield was similar between EF and VF groups, but differed among periods (*P* < 0.001) and in TWP (*P* = 0.018; Table 2). Post-hoc tests indicated that milk yield was higher by a mean of 4.9 kg per day in P0 (*P* < 0.05) and 4.6 kg per day in P1 (*P* < 0.001) than in the following periods. In addition, mean milk yield was 1.7 kg per day higher at the beginning than at the end of each period (*P* < 0.01).

### Milk cortisol

Total milk cortisol concentration ranged from 0.09 to 2.67 ng/mL, with lower values in the evening than in the morning milkings (mean ± SD: 0.55 ± 0.30 vs. 1.06 ± 0.49 ng/mL, Figure 6). The mean (±SD) concentration was 0.73 ± 0.45 ng/mL and was similar between EF and VF groups (Table 2). The LMER revealed differences among periods (*P* < 0.001) and in TWP (*P* = 0.007). Mean milk cortisol was lower during P2 to P4 compared to P0 and P1. Post-hoc tests indicated that the mean levels were by 0.15 ng/mL higher in the end than in the beginning of each period (*P* < 0.01).

## Discussion

The present study investigated the adaptation process of lactating dairy cows introduced to VF and its effects on animal welfare compared to cows managed with EF. At herd level, the total number of EPs at the virtual fence reached a peak of 18 EPs on day 1, but it decreased to five EPs after the third half-day grazing within the same paddock and remained below that threshold for the remaining part of the experiment. The cows had an average of about eight paired stimuli (AT followed by an EP) until they responded to the AT only. This result is slightly higher than, for example, Verdon et al. (2021a), who reported one virtual fence contact in 5.5 h of training, or Campbell et al. (2018b), who reported six virtual fence contacts in a feed attractant trial until animal conditioning. However, the maximum learning curve within the first three half-days of VF training was steeper than the previously identified time frames of 2 (Campbell et al., 2017) to 4 d (Lomax et al., 2019), considering that the cows in the present study were naïve to VF compared to those in the study by Campbell et al. (2017). Moreover, group sizes in Verdon et al. (2021a) were two to eight times larger than in the present study at similar paddock sizes, which may have been comparatively more conducive to social learning. Therefore, the number of fence contacts in the present study was considered comparable and within normal limits under the experimental conditions.

In week 1, the cows explored the virtual boundary for the first time and had not yet made a pairing between the AT and EP. This was reflected in a higher number of stimuli as well as a longer mean warning duration per cow compared to the remaining adaptation periods. In weeks 2 and 3 within the same paddock, there was a sharp decrease in the number of ATs and the mean warning duration, suggesting that learning was progressing and cows were recognizing when it was time to react appropriately to avoid an EP. Once the association of the paired stimuli was established, the EP became a controllable and predictable stimulus that was found to reduce signs of distress in studies by Sterling and Eyer (1988), Dougall and Baum (2011), Lee et al. (2018), and Kearton et al. (2020). The importance of a preceding acoustic cue that indicates the virtual fence has also been demonstrated in previous studies. In the absence of this warning, animals show helplessness and confusion (Lee et al., 2018), which can lead to location-based associations of the EP (Markus et al., 2014; Marini et al., 2019) or even induce misbehavior such as fear or aggression, as has been observed in dogs (Schilder and van der Borg, 2004; Blackwell et al., 2012).

With the first paddock change in the present study, the number of ATs and EPs increased again as the animals had to adapt to a new virtual boundary. However, this increase was lower than at the beginning of P1, indicating that the cows were able to apply their gained knowledge to a new virtual boundary. In P3, only two EPs were triggered at the latter part of the period and in the shorter period of P4, no EPs were recorded while ATs were still triggered, indicating that it took the cows two introductions to a new fence line to learn the system. The appropriate learning effect was also reflected by a decreasing ratio of EP/AT during the first three half-days of grazing after VF activation, as well as with each paddock change. However, we found individual differences in the rate of learning among cows, as also highlighted by previous studies (Campbell et al., 2018a; Lomax et al., 2019; McSweeney et al., 2020; Aaser et al., 2022). Some animals may require more experience at the virtual boundary due to their individual cognitive skills. The extent to which age affects the learning process in VF has not yet been scientifically proven. The study by Verdon and Rawnsley (2020) is unique in examining this aspect during a 5-d feed attractant trial with dairy heifers. Their results suggest a faster adaptation in VF of older animals (i.e., 22 mo compared to <12 mo), which, however, is in contrast to Kovalčik and Kovalčik (1986) who found a decrease in learning ability in older animals (i.e., 15-mo-old heifers vs. cows at first lactation vs. cows after second lactation) unrelated to VF. On the other hand, some animals may be pushed to test the virtual fence line by social interaction or may challenge contact at the virtual boundary themselves due to personality traits or motivation (Keshavarzi et al., 2020).

Despite individual differences in the number of fence contacts until conditioning, the cows learned to handle the VF stimuli better over time. In P1, cows showed stress-related behavioral responses after receiving an EP, reflected in running away from the virtual boundary. Cows in the present study have behaved more calmly with increasing experience in the VF system, as indicated by a higher number of retreats in P2 and P3. In addition, the cows generally kept a greater distance from the exclusion zones during P2 to P4 than during P0 and P1, but still explored the virtual fence, as shown by a high number of ATs. This further indicates that the cows learned to deal with the stimuli with more composure.

Moreover, the behavioral observations revealed a significantly higher number of displacements in the VF groups, especially in P1 when the VF system was activated for the first time. Aggressive behaviors may be triggered by frustration, which occurs when the animal is unable to effectively cope with a stressor and consequently does not achieve the expected level of environmental control (Bracke and Hopster, 2006; Špinka and Wemelsfelder, 2011; Polsky and von Keyserlingk, 2017). However, the number of displacements observed in the present study was also higher in the VF groups in P0 with the same treatment as the EF groups, but without a significant effect. Also, the number of chases in P1 was slightly higher in the VF groups than in the EF groups. However, chases were generally observed in small numbers throughout the experiment and in both, the experimental and the control groups, indicating low relevance.

Furthermore, previous research has shown that vocal behaviors in livestock may indicate stress and is therefore a valuable, noninvasive indicator of assessing animal welfare (Grandin, 1998; Manteuffel et al., 2004; Düpjan et al., 2008). For example, dairy cows that are socially isolated and/or in an unfamiliar environment are more likely to vocalize, defecate, and/or urinate (Rushen et al., 1999, 2001). In our study, the number of eliminations and vocalizations was similar in the EF and VF groups throughout the experiment, suggesting that the VF cows were not experiencing more stress than the EF cows during behavioral observations. However, as there were only a few contacts with the virtual fence during the 2-h observation periods, only a small number of recordings could be obtained in general, so the statistical results should be interpreted with caution. Furthermore, considering that the highest learning curve occurred during the first three half-days, analog to the highest number of fence contacts, the observation period should be extended, especially during the first days of an activated virtual boundary, to properly verify the behavioral responses of cows during the learning process to VF.

The spatial analysis of land use showed that cows stayed at a relative distance of about 42% of the possible maximum distance from the exclusion zones throughout the experiment. In P1, when most fence contacts were recorded, cows remained at a similar distance from the exclusion zone as in P0 with the virtual fence deactivated. In addition, cows stayed closer to the exclusion zone at the beginning of P1. Interestingly, the EP/AT rate was also highest in week 1. This could be due to the fact that the cows were exploring their grazing area and virtual boundary. In weeks 2 and 3 within the same paddock, the cows kept a greater distance from the exclusion zone, although this effect was observed equally in the EF and VF groups. Cows of the VF group still had fence contacts, as shown by a higher number of ATs, indicating that they were not afraid of approaching the virtual boundary.

Furthermore, lying time was within the range previously observed in pasture-based cows at 9 to 11 h/d (Phillips and Rind, 2001; Tucker et al., 2007). MI did not change significantly among periods but was higher at the beginning than at the end of each period. This could be related to the exploration phase of the cows as they discovered their new grazing area and fence line when changing paddocks.

The intake of NEL in the barn and the body weight showed slight fluctuations between and within the periods during the study but without noticeable irregularities. There was no decreasing trend in NEL intake, neither within nor between experimental periods. This fits well with the results for body weight, which increased over the course of the study.

Milk production was found to follow a regular diurnal pattern, with higher yields in the morning than in the evening, consistent with other studies (Gilbert et al., 1973; Quist et al., 2008). Similarly, milk cortisol concentrations showed a diurnal pattern with lower levels in the evening than in the morning, corresponding to the natural course observed in cows (Gygax et al., 2006). Previous studies have found that milk cortisol levels correlate closely with those in blood, reflecting a period of 2 (Verkerk et al., 1998) to 4 h after an acute stressor (Gellrich et al., 2015), thus making milk cortisol a useful biomarker for short- to medium-term environmental challenges (Poscic et al., 2017). In the present study, milk cortisol concentrations were relatively low but still within a range previously observed in dairy cows (Gellrich et al., 2015). In addition, cortisol levels were similar in the EF and VF groups. Furthermore, cortisol levels in the VF groups at P0 (deactivated VF) were similar to those at P1, where we expected the strongest response, and also in the subsequent adaptation periods when the cows were still receiving ATs and EPs. This indicates that the activated VF system did not cause increased stress to the cows.

All of the indicators measured showed variation among and within experimental periods, however, within normal limits. As we found no significant differences between EF and VF groups throughout the study, their variations may likely be an effect of an increased grazing time per paddock and its associated reduced forage availability on the pasture (Supplementary 1) rather than an indication of increased stress in the VF animals. The results of this study are consistent with previous studies that found no significant welfare impairment in cattle associated with the use of a VF system (Verdon et al., 2021a, 2021b; Hamidi et al., 2022a). This may be supported by the fact that the animals learn the associations of the paired stimuli of the VF system within a short period of time or a few fence contacts, making the EP controllable and predictable (Lee et al., 2018; Kearton et al., 2020), and ultimately the VF technology comparable to EF.

## Conclusion

The application of a VF system raises concerns about animal welfare due to its principle of using EPs to condition animals to an AT. The present study followed the learning process of dairy cows under VF with changing virtual boundaries and investigated possible effects on cow welfare compared to cows kept with electric fences. All cows learned to cope with the VF system within the 56 days of its activation. Most ATs and EPs occurred on the first three half-days of VF grazing. After that, the number of EPs remained low, even when a new fence line was introduced when changing paddocks. It took an average of about 8 (ranging between 3 and 11) paired stimuli until the cows responded to the AT only. Two boundary changes were sufficient to condition the cows at herd level. At the beginning of the learning phase, the cows initially showed short-term stress-related behavioral responses after receiving an EP. However, during the 2 h of observations, the cows only had few contacts with the virtual fence. Consequently, only a small number of observations was obtained and thus the statistical results have to be interpreted cautiously. Activity and lying behavior, NEL intake in the barn, body weight, milk yield, and milk cortisol concentrations were similar between virtually and electrically fenced cows. Therefore, our results suggest that the application of VF did not negatively affect animal welfare during the period studied.

## Supplementary Material

skae024_suppl_Supplementary_Material

## Abbreviations

AT: audio tone
B: beginning of each experimental period
E: end of each experimental period
EF: electric fencing
EF1 and EF2: electric fencing control group 1 and 2
EP: electric pulse
GLMM: generalized mixed effects model
GNSS: global navigation satellite systems
LMER: linear mixed effects model
LRT: likelihood ratio test
M: middle of each experimental period
MI: motion index
NEL: net lactation energy
P0: period 0
P1: period 1
P2: period 2
P3: period 3
P4: period 4
RPM: rising plate meter
TMR: total mixed ration
TWP: time within periods
VF: virtual fencing
VF1 and VF2: virtual fencing treatment group 1 and 2
