# Supplementary material for: Stress indicators in dairy cows adapting to virtual fencing
Source: J Anim Sci. 2024 Jan 25;102:skae024. doi: 10.1093/jas/skae024 (PMC10889741; doi:10.1093/jas/skae024)
Supplement: skae024_suppl_Supplementary_Material [file skae024_suppl_supplementary_material.docx]

# SUPPLEMENTARY

**Supplementary 1**: Results of the grass measurements calculated from the overall number of sampling points within the inclusion zone (**IZ**) and exclusion zone (**EZ**) at the beginning and end of each period. Results are averaged across 4 paddocks, respectively. RPM (electronic rising plate meter) clicks indicate 0.5 cm intervals of grass height. Forage yield was estimated using the formular* of Teagasc (2017) considering a post-grazing residue of 4 cm.

| **Period** | **Time Point** | **ø RPM clicks IZ** (2 x 100 RPM drops) | **ø RPM clicks EZ** (1 x 50 RPM drops) | **ø Grass height IZ (cm)** | **ø Grass height EZ (cm)** | **Estimated Forage yield*** **(kg DM / ha)** |
| --- | --- | --- | --- | --- | --- | --- |
| P0 | Start | 20.5 | 20.1 | 10.2 | **10.0** | 1558 |
| P0 | End | 21.3 | 19.3 | 10.6 | 9.6 | 1658 |
| P1 | Start | 21.3 | 19.3 | 10.6 | 9.6 | 1658 |
| P1 | End | 15.2 | 20.2 | 7.6 | 10.1 | 894 |
| P2 | Start | 11.4 | 11.4 | 5.7 | 5.7 | 425 |
| P2 | End | 10.1 | 13.3 | 5.1 | 6.7 | 264 |
| P3 | Start | 15.4 | 15.9 | 7.7 | 7.9 | 914 |
| P3 | End | 14.2 | 19.5 | 7.1 | 9.7 | 770 |
| P4 | Start | 13.7 | 15.5 | 6.8 | 7.7 | 708 |
| P4 | End | 12.0 | 16.0 | 6.0 | 8.0 | 502 |

*$Forage yield ({kg DM}/{ha)=\left( height-4 cm \right)\times250 {kg DM}/{cm}}$
